# Effect of Ultrasonic Penetration with Volatile Oil of Olibanum and Chuanxiong Rhizoma on Acute Knee Synovitis Induced by Sports Training: An Open-Label Randomized Controlled Study

**DOI:** 10.1155/2022/6806565

**Published:** 2022-02-28

**Authors:** Qiwen Xuan, Yi Ruan, Chengbing Cao, Zifei Yin, Juan Du, Can Lv, Fanfu Fang, Wei Gu

**Affiliations:** ^1^School of Traditional Chinese Medicine, Naval Medical University, Shanghai 200433, China; ^2^Department of Rehabilitation, Changhai Hospital, Naval Medical University, Shanghai 200433, China

## Abstract

**Background:**

Knee synovitis is a common sports injury. We proposed the use of UTV_OR_, which is a combination of the use of volatile oil of Olibanum (VOO) and volatile oil of Chuanxiong Rhizoma (VOCR) and conventional ultrasound (US) therapy, to treat knee synovitis. *Design, Setting, Participants, and Interventions*. Participants were randomly assigned into a control group (conventional US therapy group) and a test group (UTV_OR_ group). The control group received conventional US therapy with a coupling agent as the medium. The test group received a revised US therapy with VOO and VOCR as media. Both groups were treated once per day for three consecutive days. *Main Outcome Measures*. The subjects' Visual Analogue Scale (VAS) pain score, Lysholm knee score, knee swelling degree, circumference, and range of motion of the knee joint were evaluated before the first treatment and 24 h after the third treatment. The VAS pain score was considered the primary outcome, while the three other measurements were regarded as the secondary outcomes. An adverse event was reported subjectively and recorded.

**Results:**

A total of 116 participants were included in the analysis (test group: *n* = 64; control group: *n* = 52). The evaluation results showed that the VAS pain scores of the male and female participants in both groups decreased after treatment (*P* < 0.001), but only the difference among the male sub-group had significant between-group difference (*P* < 0.001). After treatment, the Lysholm scores in both groups increased significantly (all *P* < 0.001), the range of motion and the circumference of the injured knee decreased significantly (*P* < 0.001), while no between-group difference was observed in general or in the gender sub-groups (all *P* > 0.025). No side effect or complication was reported during the treatment.

**Conclusion:**

UTV_OR_ had a superior analgesic effect to conventional US therapy in the male population, but its effects on alleviating joint function, swelling, and range of motion were comparable to that of conventional US therapy. Our study found that UTV_OR_ can be an effective method to reduce pain and treat knee synovitis, and it is subjectively safe. *Trial registration*. This study was registered under the Chinese Clinical Trial Registry (Trial Registration Number: ChiCTR2000035671).

## 1. Introduction

Synovitis is a key factor in osteoarthritis pathophysiology because of the action of several soluble mediators [1]. Synovitis in osteoarthritis was believed to be a consequence of underlying joint damage, with synovial inflammation resulting in typical clinical signs such as redness, swelling, pain, or heat [1, 2].

The diagnosis of knee synovitis is closely related to knee joint pain [3]; thus, the treatment of knee synovitis is mainly aimed at pain alleviation. Physiotherapy is a type of non-pharmacological intervention for osteoarthropathy recommended by the American College of Rheumatology and the European League against Rheumatism [4]. Ultrasound (US) therapy is one of the most common physiotherapy physical agents used within physiotherapy practice in several countries for the management of acute knee synovitis [5].

Olibanum and Ligusticum chuanxiong are traditional Chinese medicines that promote blood circulation and relieve pain. Volatile oil of Chuanxiong Rhizoma (VOCR) and volatile oil of Olibanum (VOO) are low-polarity and volatile components obtained through the distillation or extraction of the traditional Chinese medicine, Ligusticum chuanxiong, and Olibanum, and these components are used as primary medicinal ingredients [6, 7].

On the basis of the property of US therapy, VOO, and VOCR, we proposed to combine VOO, VOCR, and conventional US therapy into a method called UTV_OR_ for the treatment of knee synovitis, but no systematical data have confirmed its effects prior to the current study. Although previous studies have reported that traditional Chinese medicine can be used to treat synovitis [8], the present work is the first attempt to combine traditional Chinese medicine and physical therapy to treat synovitis. This study thus aimed to observe the effect of UTV_OR_ on knee synovitis among people undergoing sports training, especially on alleviating the patient's joint swelling and pain and restoring joint movement function. We hypothesized that UTV_OR_ can treat knee synovitis better than conventional US therapy.

## 2. Methods

### 2.1. Trial Design

The current study was an open-label and randomized, controlled clinical trial (RCT) conducted in a sports training base. Participants were randomly assigned to a control group (conventional US therapy group) and a test group (UTV_OR_ group). The control group received conventional US therapy with a coupling agent as the medium. The test group received revised US therapy with VOO and VOCR as media. Both groups were treated once per day for three consecutive days. Participants, therapists, and other researchers had no communication with one another.

The study adhered to the Declaration of Helsinki. The protocol of this study was registered under the Chinese Clinical Trial Registry (Trial Registration Number: ChiCTR2000035671) prior to recruitment and approved by the sport training base. The entire research was monitored by a qualified clinical trial specialist. All treatments were conducted by experienced therapists from the Rehabilitation Department of Changhai Hospital, Shanghai, China.

### 2.2. Participants

The patients were enrolled in this RCT from October to December 2020 from a sports training base at Shanghai, China. Eligible patients were recruited and randomly assigned to either the treatment group or the control group.

Patients included in the study met the following criteria: in the sports base who were diagnosed with acute knee synovitis according to Expert Consensus on Diagnosis and Clinical Efficacy Evaluation of Adult Knee Synovitis [9]; not undergoing any treatment for knee synovitis within the last two weeks; and voluntarily participating in this study and signing an informed consent form.

The exclusion criteria were as follows: participating in other clinical trials within one month before enrollment; withdrawing their informed consent during the trial; and using other treatments during the trial.

All patients were asked to sign informed consent forms, thereby agreeing to receive either kind of treatment. The participants' personal data were kept confidential, and they were allowed to withdraw at any time during the trial without affecting any treatment. Subjects who had not expressed their desire to withdraw but did not accept the assigned treatment or missed the required follow-up were deemed withdrawn. Data from withdrawn participants were excluded. For each participant, only a unilateral knee was included in the analysis; if one participant suffered from bilateral knee synovitis, then only the more severe knee would be chosen, or the right knee would be chosen when the severity was similar. We followed the Consolidated Standards of Reporting Trials guideline for designing and reporting this trial [10].

### 2.3. Interventions

The control group received conventional US therapy as follows: first, the patient maintained a comfortable position; Sonopuls90 US therapy instrument (Enraf-Nonlus BV, Netherland) was then used for treatment with a coupling agent as medium; output frequency was 1 MHz; the treatment dose was adjusted within the range of 0.8–1.0 W/cm^2^ in the continuous mode according to the subject's condition and moved slowly around the pain point of the affected area at a speed of 2–3 cm/s for 10 min, once a day, for three consecutive days.

For the test group, UTV_OR_ was performed as follows: first, 10 drops (3 mL) of 20% mixed oil of VOO (200601, Ji'an Asus Spice Oil Co., Ltd.) and VOCR (201107, Ji'an Asus Spice Oil Co., Ltd.) were evenly smeared on the affected area; US therapy was then performed similar to that of the control group with a little coupling agent as medium. During the course of treatment, the participants in both groups were instructed to rest and minimize the amount of exercise.

### 2.4. Outcomes

The four measurements include Visual Analogue Scale (VAS) pain score, Lysholm knee score, range of motion of the injured knee, and circumference of the injured knee. Both groups were assessed before the first treatment and 24 h after the third treatment. The VAS pain score was regarded as the primary outcome, and the three other measurements were considered the secondary outcomes.

#### 2.4.1. VAS Pain Score (the Primary Outcome)

The VAS pain score adopts the pain measurement method used by the Clinical Research Center of the National Institutes of Health as described in this subsection [11]. A 10-cm horizontal line is drawn on a paper. One end of the horizontal line is 0, indicating no pain; the other end is 10, meaning severe pain; the middle part means different degrees of pain. The patient was asked to draw a mark on the horizontal line based on how they felt and to indicate the degree of pain. The following scoring was adopted: 0 points: no pain; 3 points or less: slight pain, which the patient can tolerate; 4–6 points: moderate pain, which affects sleep, but is tolerable; 7–10 points: gradually intense pain, and the pain is difficult to endure.

#### 2.4.2. Lysholm Knee Score (the Secondary Outcome)

The Lysholm knee score [12] was used to evaluate the knee joint function of patients via eight items about the following aspects: claudication, squatting, support, up stairs, pain, instability, sense of closure and swelling. The total score is 100 points. A higher score indicates better knee function.

#### 2.4.3. Range of Motion of the Injured Knee (the Secondary Outcome)

**Figure 1:**
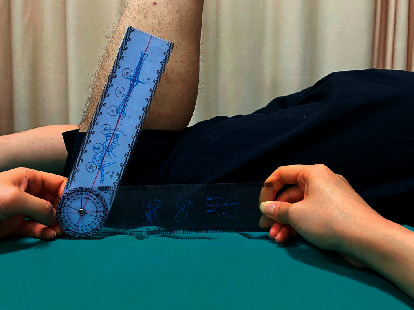
Therapist measuring the range of motion of the patient's injured knee joint.

The range of motion of the injured knee was determined as follows: first, the participant maintained a prone position with the knee joint extended; second, the goniometer's axis was placed on the knee joint, the fixed arm was attached to the femur's longitudinal axis, and the moving arm was attached to the line connected the head of the fibula and the lateral condyle, allowing the participant to flex the calf as close as possible to the thigh; finally, the degree was recorded as range of motion. Degrees ranging from 0° to 135° were considered normal. The measurement result is accurate to 0.1° (Figure 1).

#### 2.4.4. Circumference of the Injured Knee (the Secondary Outcome)

**Figure 2:**
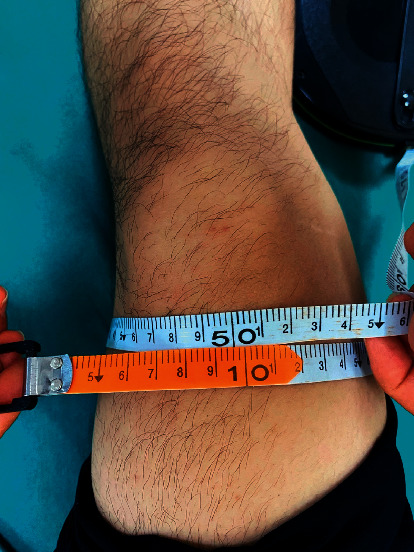
Therapist measuring the circumference of the patient's injured knee.

The circumference of the knee joint was measured where the pain was strongest and in the same manner by the same doctor each time (marked with an indelible pen, accurate to 0.1 cm). A tape was used to measure the circumference of the knee joint, with the participants lying supine. The measuring tape has been positioned above the upper edge of the patella (Figure 2).

#### 2.4.5. Harms

Participants were required to report any adverse event throughout the trial. The details of each adverse event were recorded to document the study's safety.

### 2.5. Sample Size

PASS software (Version 15.0, NCSS LLC, Utah, USA) was used to calculate the sample size on the basis of the primary outcome, the VAS pain score. We set the expected mean ± standard deviation as 1.5 ± 1.0 for the test group and 2.2 ± 1.0 for the control group according to a previous study [13]. The required sample size was thus calculated to be 52 patients in each group. Assuming an expected drop-out rate of 20%, a total number of 66 patients for each group would be randomized to each group.

### 2.6. Randomization, Allocation Concealment Mechanism, and Implementation

Patients who were interested in participating in this trial were first interviewed. The randomization sequence was generated via SPSS (Version 25.0; SPSS Inc., Chicago, IL, USA) by an independent researcher and placed in opaque sealed envelopes to ensure the allocation concealment. Of the 132 random numbers, 1–66 were set as the test group, and 67–132 were set as the control group. Eligible subjects who provided written informed consent were randomly assigned to a group. The envelopes were managed by an independent, blinded statistician who was not involved in participant recruitment, treatment, or assessment. Before the first treatment, the therapist opened the envelope of each eligible patient to determine which group they entered.

### 2.7. Blinding

This was an open-label study. Therefore, participants and intervention providers were not blinded. However, the researchers conducting data analysis were blinded for the group intervention to avoid bias of assessment.

### 2.8. Statistical Methods

The outcomes were analyzed on the basis of Per Protocol Set as follows: only participants who underwent all of the assigned interventions were included in the analysis. The safety was analyzed on the basis of the safety set.

All data were entered twice by two researchers. Statistical analysis was conducted via SPSS (Version 21.0, IBM Corp., Armonk, New York, USA). Measurement data were expressed as the mean ± standard deviation; the normality of data was tested via Shapiro–Wilk test; within-group comparisons were performed using the paired *t*-test or Wilcoxon signed rank test for normality and non-normality data, respectively; and between-group differences were compared using Student's *t*-test or Mann–Whitney *U* test. Categorical data were expressed as number and percentage (%), and between-group comparisons were performed using chi-squared test. A *P* value <0.05 for demographic data was considered statistically significant. Considering that within- and between-group differences for outcome measurements should be simultaneously tested to assess the effect of time and intervention together, the Bonferroni correction method was used, and the level of significance was reduced to 0.05/2 = 0.025. Studies have shown that due to differences in physiological factors and training methods, female participants are twice as likely to be injured as males. Thus, we performed a subgroup analysis by gender [14].

## 3. Results

### 3.1. Participant Flow, Recruitment, and Baseline Data

**Figure 3:**
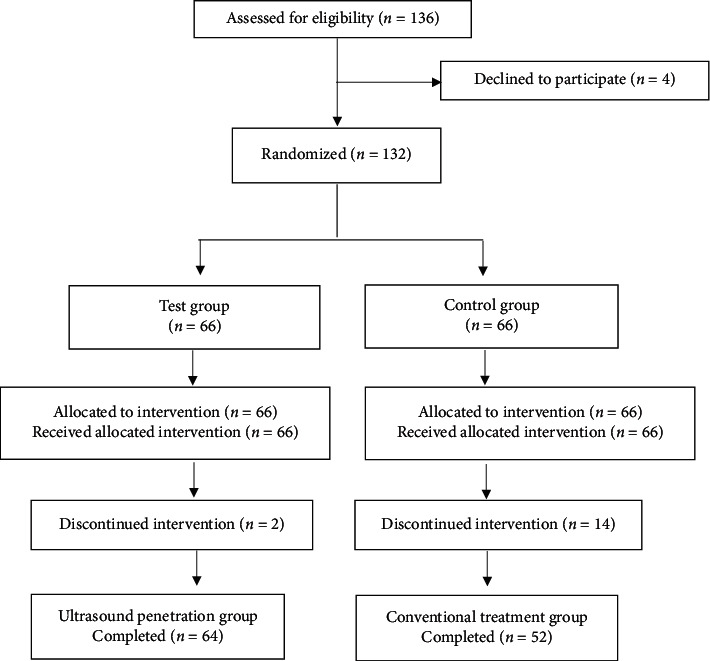
Flow chart of this study. The control group received conventional US therapy. The test group received a US therapy with the coupling agent substituted by VOO and VOCR as media.

From October to December 2020, a total of 136 participants were enrolled in this study. Four enrolled patients then voluntarily withdrew from the study before the treatment. Hence, 132 patients were successfully randomized to the control and test groups. During the study, two and fourteen patients in the test and control groups discontinued intervention due to the training assignment, respectively. Finally, 64 participants in the test group and 52 in the control group were included in the analysis. The study flow chart is shown in Figure 3.

As shown in Table 1, the height, weight, and body mass index (BMI) were normally distributed (*P* > 0.05), while age was not normally distributed (*P* < 0.05). The demographic characteristics of both groups showed that the age, height, weight, and BMI were comparable (all *P* > 0.05; Table 2). The average age was 22.05 ± 2.86 years in the control group and 21.90 ± 2.30 years in the test group. BMI was found to be 22.25 ± 2.10 in the test group and 22.96 ± 2.42 in the control group. The number of male patients in both groups was higher than the number of female patients (62.5% *versus* 51.6% in the test group; 78.8% *versus* 21.2% in the control group), but the gender composition had no significant between-group difference (*P*=0.069). In addition, no between-group difference was observed in the lateral of injured knees (*P*=0.264). The majority of the participants had no or slight swollen knee (82.8% in the test group and 88.5% in the control group). Still, no between-group difference was observed in the swelling degree of the injured knee (*P*=0.610).

### 3.2. VAS Pain Score

The VAS pain scores of both groups during the treatment are shown in Table 3. In general, the VAS pain scores of both groups decreased significantly after receiving treatment (both within-group *P* values <0.001), but the VAS pain scores of the test group were significantly lower than those of the control group after treatment, when compared with the baseline (1.11 ± 1.32 *versus* 2.13 ± 1.50; between-group *P* value <0.001). The results of the sub-group analysis by gender showed that the two groups had the same effect of reducing VAS pain scores in men and women. The VAS pain scores of men and women decreased significantly after treatment (all within-group *P* values <0.001). However, only the VAS pain score in the male sub-group had significant between-group difference between the test and control groups after treatment (1.00 ± 1.32 *versus* 2.22 ± 1.56, between-group *P* value <0.001). No corresponding between-group difference was observed in the female sub-group (between-group *P* value = 0.158).

### 3.3. Lysholm Score

The Lysholm scores are shown in Table 4. Generally, the Lysholm scores of both groups increased significantly after treatment (both within-group *P* values <0.001) but had no significant between-group difference (*P*=0.156 > 0.025). The results of subgroup analysis based on gender classification showed similar results (all between-group *P* values >0.025).

### 3.4. Range of Motion of the Injured Knee

In general, the range of motion of the injured knee joints of the two groups improved significantly after treatment (both within-group *P* values <0.001), but no significant between-group difference was observed (Table 5). The results of the subgroup analysis according to gender classification had the same results.

### 3.5. Circumference of the Injured Knee

In general, the circumference of the injured knee joint of both groups decreased significantly after treatment (both within-group *P* values <0.001), but no significant between-group difference was observed (Table 6). The results of subgroup analysis according to gender classification had the same results.

### 3.6. Harms

No side effect or complication was reported during the treatment.

## 4. Discussion

### 4.1. Interpretation

The synovium is a membrane attached to the skeletal tissue at the bone-cartilage interface that borders the joint cavities and that lines tendon sheaths and bursae. Inflamed synovial tissue exhibits the following three major histological alterations: hyperplasia of the intimal lining layer due to accumulation of macrophages and proliferation of fibroblast-like synoviocytes that are in an altered activation state; neoangiogenesis with endothelial activation in the synovial sublining; and a vast influx of inflammatory cells. These infiltrating leucocytes are activated and produce a vast amount of pro-inflammatory and destructive mediators that contribute to synovitis and to cartilage and bone destruction [15]. A palpable joint swelling in an “inflamed” synovium is considered to be due to the thickening of the synovium (through the development of pannus) or synovial fluid effusion [1]. Synovitis is directly responsible for several clinical symptoms and reflects the structural progression of osteoarthritis [1].

At present, non-steroidal anti-inflammatory drugs are widely used to alleviate the pain and stiffness associated with acute knee synovitis, but side effects such as severe upper gastrointestinal limit the use of these drugs [16]. Alternative means of treatment that are environment friendly and able to stop inflammation, pain, and degeneration of the joint cartilage in osteoarthritis patients urgently need to be developed [17]. US treatment has been used as a non-invasive modality for the management of osteoarthritis for more than 60 years because of its reputed ability to relieve pain, reduce edema, increase the range of motion, and accelerate tissue repair via thermal and non-thermal mechanisms (mechanical effects) [13]. A systematic review and meta-analysis suggested that pulsed US is the preferred treatment mode in terms of effective pain relief and improved function without significant adverse effects in clinical trials [18]. A previous study examined the potential of low-intensity pulsed US as an effective approach for treating the inflammatory activity of synovitis [19]. Their studies showed that low-intensity pulsed US significantly suppresses cell proliferation and growth and the DNA fragmentation (a feature of apoptosis) of synovial membrane HIG-82 cells simulated by cytokine TNF-*α*, IL-1*β*, iNOS, and chemoattractant chemokine receptor CCR5 [18].

Olibanum was an approved remedy in Europe against inflammation at the beginning of the 20th century and is mentioned in the seventh supplement of the European Pharmacopoeia from 2006. Oleogum resins and essential oils of Olibanum exert anti-inflammatory effects. Its essential oils consist of monoterpenes and aliphatic molecules. The underlying molecular mechanisms include the activation of antioxidant enzymes, anti-inflammatory properties, hepatic enzymes, the activation of intracellular signaling routs, and transcription factors regulating gene expression. Pro-inflammatory cytokines are downregulated, whereas anti-inflammatory cytokines are upregulated [20]. Clinical studies have shown that an Olibanum extract not only has anti-inflammatory and anti-arthritis properties but also improves pain and physical functions [21]. Dozens of compounds have been detected in Ligusticum chuanxiong, and the major compounds were ligustilide and butylidenephthalide with relative contents of 67.46% and 5.06%, respectively [22]. Ligustilide was also identified as efficacious pregnane X receptor agonist [23], which may be the reason for its anti-inflammatory effect [6]. VOCR can significantly raise the pain threshold of mice in the hot-plate test and reduce the number of writhing response caused by chemical materials, demonstrating its central analgesic effect. The mechanism of analgesic effects may be related to adjusting the level of opioid peptide in the central nervous system by affecting *c-fos* gene expression in the brain tissue, raising the levels of 5-hydroxytryptamine and endothelin, and reducing calcitonin gene-related peptide level in plasma [24]. Studies have shown that VOCR can increase the pain threshold and has various medicinal properties such as analgesia and anti-inflammatory effects [6]. Another research revealed that VOO helps in promoting the permeability of VOCR from the epidermis to dermal capillaries by increasing skin blood flow, thus enhancing the transdermal permeation amounts of drugs [25].

This study was a RCT that combined traditional Chinese medicine with rehabilitation equipment in the treatment of acute knee synovitis patients and provides a new method for the treatment of acute knee synovitis. The results of this study (Tables 3to 6) revealed that after treatment, the VAS pain score and the circumference of the injured knee significantly decreased; the Lysholm score and the range of motion of the injured knee significantly increased in both groups, indicating that both the conventional US therapy and UTV_OR_ can improve the pain, swelling, joint function, and range of motion caused by knee synovitis. Further, the between-group comparison demonstrated that in the test group, the VAS pain score decreased further in the male sub-group, the Lysholm score increased significantly in both genders, while the range of motion and the circumference of the injured knee were comparable. These findings imply that UTV_OR_ had a superior analgesic effect than conventional US therapy in the male population. In addition, UTV_OR_ has the advantage of improving the joint function without gender difference but has no effect in alleviating joint swelling and improving the range of motion in knee synovitis.

Pain tolerance is different for each patient; therefore, measuring pain levels using the VAS pain score inevitably causes a subjective bias [26]. The gender-different outcomes of UTV_OR_ on pain alleviation may be explained by the higher sensitivity and poorer tolerability of females to pain than that of males, resulting in a strong placebo effect [27, 28]. The gender-difference is also possibly due to other human factors, such as work pressure and social environment, which causes females and males to express pain differently. These human factors can also explain why females' conditions are more severe than those of males on the basis of the comparison of scores before treatment. Although we only conducted physical therapy in the treatment process, psychological counseling was often performed during the treatment. Psychological counseling may be more effective for females, which may explain why no significant difference was observed after the treatment. Participants were asked to minimize physical exercise during the treatment. Aside from affecting their own treatment effects, the patient's pain cannot tolerate high-intensity exercise. The factor of rest also possibly plays a positive role in the patient's recovery from the disease.

In the past, clinical trials were mostly aimed at the use of US for diagnosis or positioning. The present study changed the conventional US medium (i.e., coupling agent) to essential oils of traditional Chinese medicine for the first time, and explored the therapeutic effect of combining US therapy with volatile oils of traditional Chinese medicine on the treatment of acute knee synovitis caused by sports training. The proposed method was found to significantly improve joint pain and swelling and restore the joint movement function with no extra adverse reactions. US introduction of VOR and VOO is an efficient, easy-to-operate, and non-invasive treatment, which opens up novel methods for clinical treatment.

### 4.2. Generalisability

This study provides support for the effect of UTV_OR_ by popularization and application and also confirms the timeliness of the effects of a few (i.e., three times) treatments. Moreover, the proposed method is more adapted to the demand for rapid curative effects in the treatment of sports training injuries and provides a reference for better short-term application of US technology to treat related diseases. In this study, the combination of US therapy and volatile oils of traditional Chinese medicine for treatment is an innovative point of integration of traditional Chinese and Western medicine. The combination is more effective. During the treatment process, volatile oil was also found to have an evident diathermy effect, which makes the injured knee joint of the participants feel warm and comfortable.

### 4.3. Limitations

This study had several limitations. The curative effect observation index selected in this study was relatively simple, and the objective index, such as nuclear magnetic field, was not selected; therefore, the effect of improving the amount of intra-articular fluid cannot be confirmed. In addition, the participants in this study are relatively single in type, and more extensive research should be considered in the future. Considering that no reference in the relevant literature was made, determining whether the volatile oil will affect the penetration of US is impossible. The experimental group in this experiment chose to add volatile oil considering the small amount of coupling agents, and the molecular mechanism of its permeability can be further improved at a later time. In addition, the addition of a topical volatile oil smear group should be considered for observation and control in a more extensive study to further confirm the effect of volatile oil.

## 5. Conclusion

This study showed that the ultrasonic therapy instrument penetrating the volatile oil of traditional Chinese medicine could be an effective treatment method, which can effectively reduce the pain of knee synovitis patients caused by sports training injuries and improve knee function, thereby improving training efficiency. Besides, its safety has been confirmed at least in the subjective perspective.

## Figures and Tables

**Table 1:** Normality test results.

Item	Test group (*n* = 52)			Control group (*n* = 64)		
	Statistic	d*f*	*P* value	Statistic	d*f*	*P* value
Age	0.795	62	<0.001	0.883	52	<0.001
Height	0.975	62	0.230	0.975	52	0.348
Weight	0.973	62	0.185	0.967	52	0.150
BMI	0.976	62	0.268	0.960	52	0.081
VAS						
Before-treatment	0.849	62	<0.001	0.872	52	<0.001
After-treatment	0.675	62	<0.001	0.920	52	0.002
Lysholm score						
Before-treatment	0.947	62	0.009	0.904	52	0.001
After-treatment	0.825	62	<0.001	0.825	52	<0.001
Range of motion of the injured knee						
Before-treatment	0.845	62	<0.001	0.875	52	<0.001
After-treatment	0.967	62	0.089	0.805	52	<0.001
Circumference of the injured knee						
Before-treatment	0.972	62	0.163	0.905	52	0.001
After-treatment	0.978	62	0.336	0.881	52	<0.001

BMI: body mass index; VAS: visual analogue scale. The normality was assessed via Shapiro–Wilk test.

**Table 2:** Demographic characteristics of both groups (*N* = 116).

Characteristics	Test group (*n* = 64)	Control group (*n* = 52)	*P* value
Age (years, mean ± SD)	22.05 ± 2.86	21.90 ± 2.30	0.906^a^
Height (cm, mean ± SD)	172.66 ± 7.12	171.83 ± 6.44	0.516^b^
Weight (kg, mean ± SD)	66.78 ± 9.59	68.08 ± 10.07	0.481^b^
BMI (kg/m^2^)	22.25 ± 2.10	22.96 ± 2.42	0.095^b^
Gender (*n*[%])			
Male	40 (62.5)	41 (78.8)	0.069^c^
Female	24 (37.4)	11 (21.2)	
Injured knee (*n*[%])			
Left	33 (62.5)	21 (40.4)	0.264^c^
Right	31 (51.6)	31 (59.6)	
Swelling degree of the injured knee			
No	26 (40.6)	26 (50.0)	0.610^c^
Slight	27 (42.2)	20 (38.5)	
Moderate	10 (15.6)	6 (11.5)	
Severe	1 (1.6)	0 (0)	

SD: standard deviation. ^a^The between-group *P* value was calculated by Mann–Whitney *U* test for “age.” ^b^The between-group *P* value was calculated by Student *t*-test for “height,” “weight,” and “BMI.” ^c^The between-group *P* value of *n* [%] was calculated by chi-squared test.

**Table 3:** VAS pain scores of both groups (*N* = 116).

Items	Test group (*n* = 64)	Control group (*n* = 52)	Between-group *P* value
All participants (*n* = 116)			
Before-treatment	4.14 ± 1.27	3.88 ± 1.06	0.368
After-treatment	1.11 ± 1.32	2.13 ± 1.50	<0.001^#^
Within-group *P* value	<0.001^#^	<0.001^#^	—
Male (*n* = 81)			
Before-treatment	3.98 ± 1.12	3.80 ± 1.03	0.525
After-treatment	1.00 ± 1.32	2.22 ± 1.56	<0.001^#^
Within-group *P* value	<0.001^#^	<0.001^#^	—
Female (*n* = 35)			
Before-treatment	4.42 ± 1.47	4.18 ± 1.17	0.767
After-treatment	1.29 ± 1.33	1.82 ± 1.25	0.158
Within-group *P* value	<0.001^#^	<0.001^#^	—

The between-group *P* value was calculated by Mann–Whitney *U* test. The within-group *P* value was calculated by Wilcoxon signed rank test. ^#^The *P* value was statistically significant under the adjusted significant level as 0.025 based on Bonferroni correction. The test group included 40 males and 24 females, and the control group included 41 males and 11 females.

**Table 4:** Changes in Lysholm scores in both groups (*N* = 116).

Items	Test group (*n* = 64)	Control group (*n* = 52)	Between-group *P* value
All participants (*n* = 116)			
Before-treatment	72.23 ± 14.11	75.67 ± 16.34	0.080
After-treatment	90.33 ± 10.10	86.04 ± 14.26	0.156
Within-group *P* value	<0.001^#^	<0.001^#^	—
Male (*n* = 81)			
Before-treatment	76.08 ± 12.47	75.95 ± 15.75	0.723
After-treatment	91.23 ± 11.83	85.10 ± 15.65	0.036
Within-group *P* value	<0.001^#^	<0.001^#^	—
Female (*n* = 35)			
Before-treatment	65.83 ± 14.62	74.64 ± 19.21	0.031
After-treatment	88.83 ± 6.15	89.55 ± 8.32	0.914
Within-group *P* value	<0.001^#^	<0.001^#^	—

The between-group *P* value was calculated by Mann–Whitney *U* test. The within-group *P* value was calculated by Wilcoxon signed rank test. ^#^The *P* value was statistically significant under the adjusted significant level as 0.025 based on Bonferroni correction. The test group included 40 males and 24 females, and the control group included 41 males and 11 females.

**Table 5:** Range of motion of the injured knee (*N* = 116).

Items	Test group (*n* = 64)	Control group (*n* = 52)	Between-group *P* value
All participants (*n* = 116)			
Before-treatment	109.95 ± 13.22	111.65 ± 11.80	0.586
After-treatment	118.30 ± 7.18	116.42 ± 11.50	0.760
Within-group *P* value	<0.001^#^	<0.001^#^	—
Male (*n* = 81)			
Before-treatment	110.81 ± 12.96	110.8 ± 12.32	0.806
After-treatment	118.18 ± 6.04	115.69 ± 12.42	0.741
Within-group *P* value	<0.001^#^	<0.001^#^	—
Female (*n* = 35)			
Before-treatment	108.53 ± 13.82	114.81 ± 9.42	0.189
After-treatment	118.50 ± 8.90	119.14 ± 6.90	0.972
Within-group *P* value	<0.001^#^	<0.001^#^	—

The between-group *P* value was calculated by Mann–Whitney *U* test. The within-group *P* value was calculated by Wilcoxon signed rank test. ^#^The *P* value was statistically significant under the adjusted significant level as 0.025 based on Bonferroni correction. The test group included 40 males and 24 females, and the control group included 41 males and 11 females.

**Table 6:** Circumference of the injured knee (*N* = 116).

Items	Test group (*n* = 64)	Control group (*n* = 52)	Between-group *P* value
All participants (*n* = 116)			
Before-treatment	36.32 ± 2.68	36.54 ± 2.83	0.758
After-treatment	35.88 ± 2.54	36.15 ± 2.79	0.822
Within-group *P* value	<0.001^#^	<0.001^#^	—
Male (*n* = 81)			
Before-treatment	36.34 ± 2.89	36.84 ± 2.93	0.490
After-treatment	36.12 ± 2.78	36.56 ± 2.89	0.620
Within-group *P* value	<0.001^#^	<0.001^#^	—
Female (*n* = 35)			
Before-treatment	36.29 ± 2.35	35.44 ± 2.20	0.213
After-treatment	35.49 ± 2.08	34.6 ± 1.69	0.131
Within-group *P* value	<0.001^#^	<0.001^#^	—

The between-group *P* value was calculated by Mann–Whitney *U* test. The within-group *P* value was calculated by Wilcoxon signed rank test. ^#^The *P* value was statistically significant under the adjusted significant level as 0.025 based on Bonferroni correction. The test group included 40 males and 24 females, and the control group included 41 males and 11 females.

## Data Availability

The data used to support the findings of this study are included within the article.

## Supplementary Materials

Supplementary Materials Numbers refer to the serial number; Groups refer to the test/control groups; Leg refers to which leg the injured knee belongs to; Age, Height, Weight, and BMI are the real information of the participants; Visual Analogue Scale pain score was regarded as the primary outcome; and the Lysholm, Range of Motion, Circumference were considered the secondary outcomes. Both groups were assessed before the first treatment (referred to before in the picture) and 24 h after the third treatment (referer to after in the picture).
