# Supplementary material for: Effect of Ultrasonic Penetration with Volatile Oil of Olibanum and Chuanxiong Rhizoma on Acute Knee Synovitis Induced by Sports Training: An Open-Label Randomized Controlled Study
Source: Pain Res Manag. 2022 Feb 28;2022:6806565. doi: 10.1155/2022/6806565 (PMC8901337; doi:10.1155/2022/6806565)

**Supplementary descriptions**

Numbers refer to the serial number;

Groups refer to the test/ control groups;

Leg refers to which leg the injured knee belongs to;

Age、Height、Weight、BMI are the real information of the participants;

Visual Analugue Scale pain score was regarded as the primary outcome, and the Lysholm、Range of Motion、Circumference were considered the secondary outcomes. Both groups were assessed before the first treatment（refered to before in the picture）and 24 h after the third treatment（referer to after in the picture）.


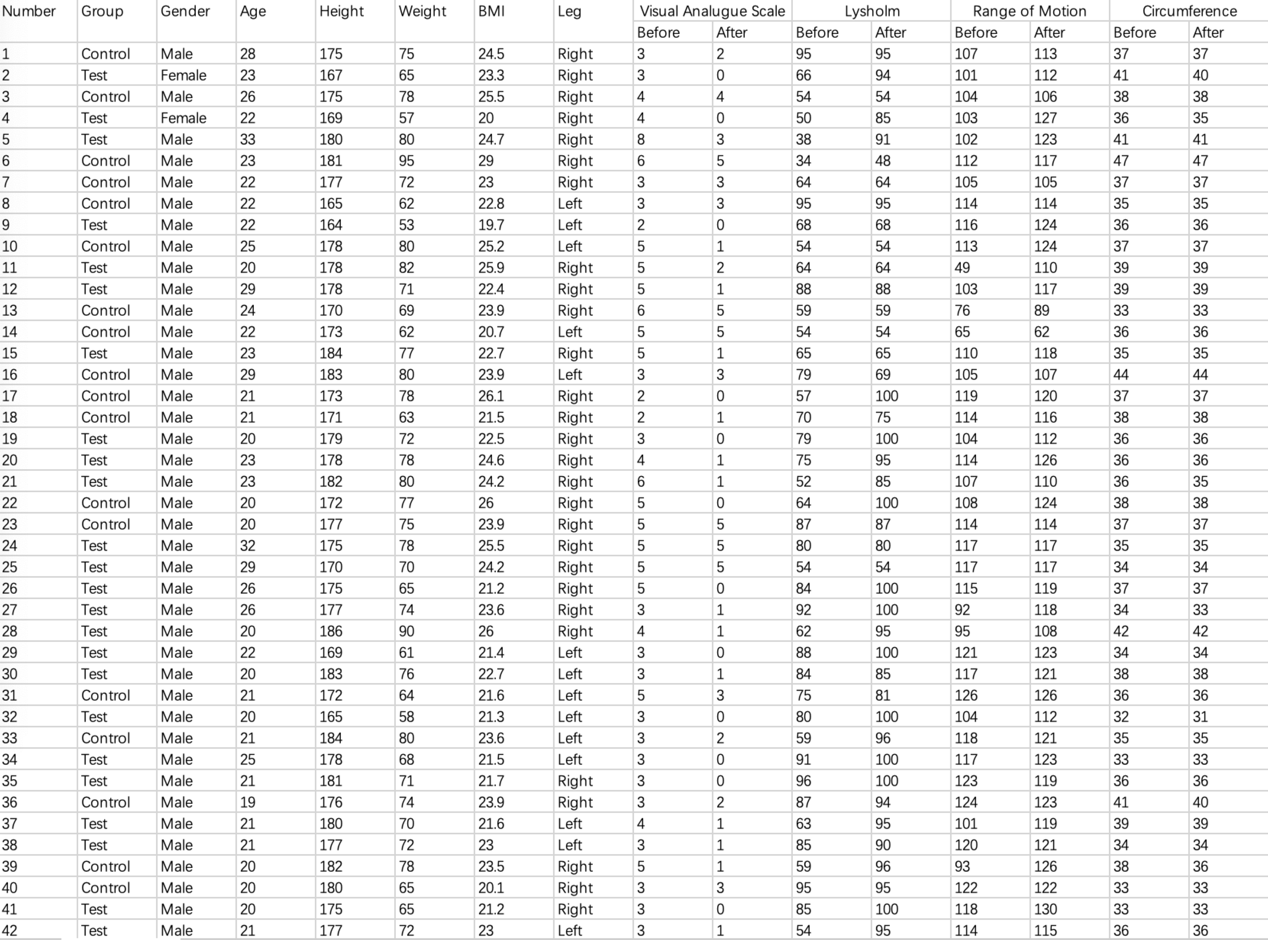


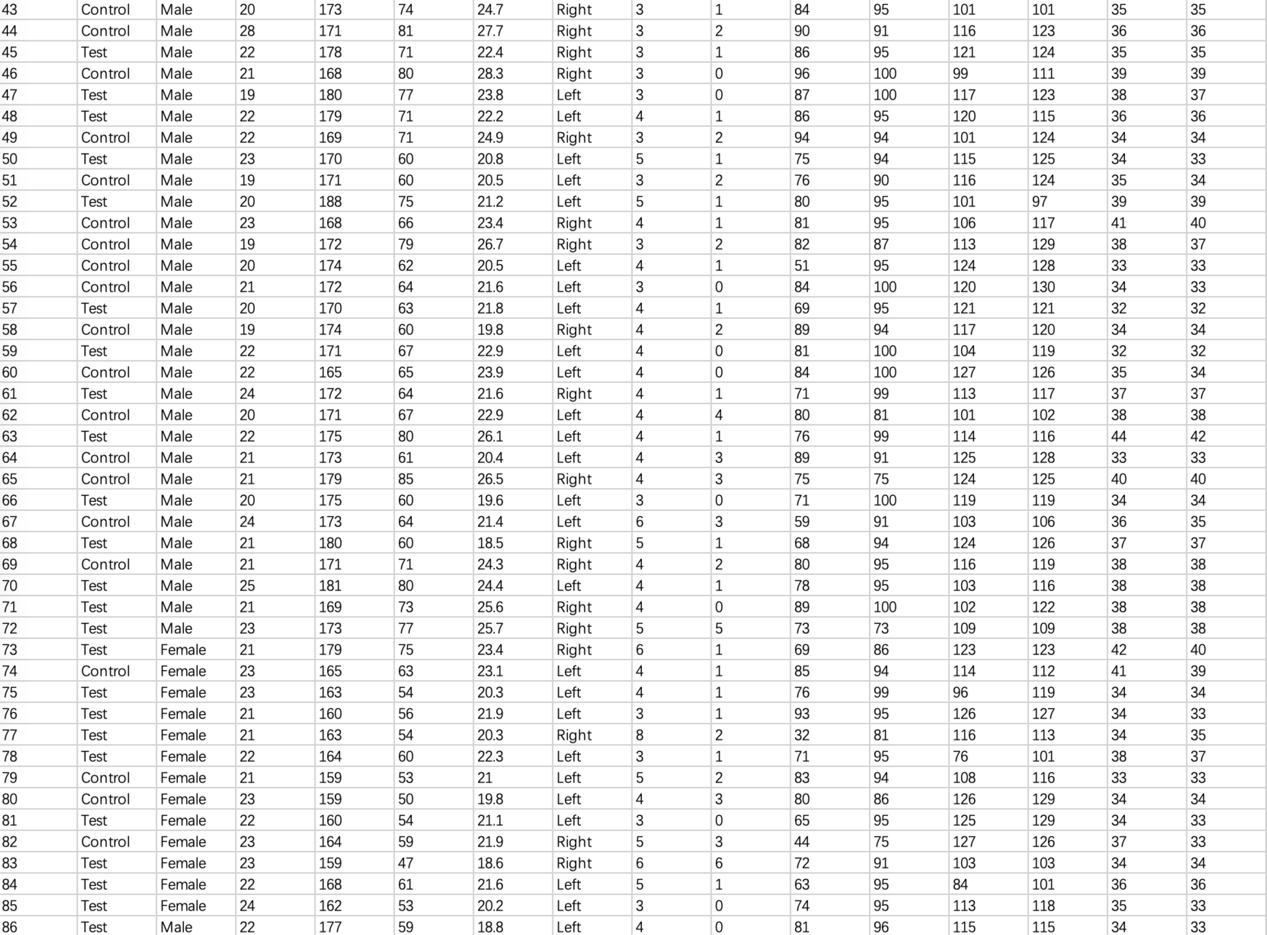


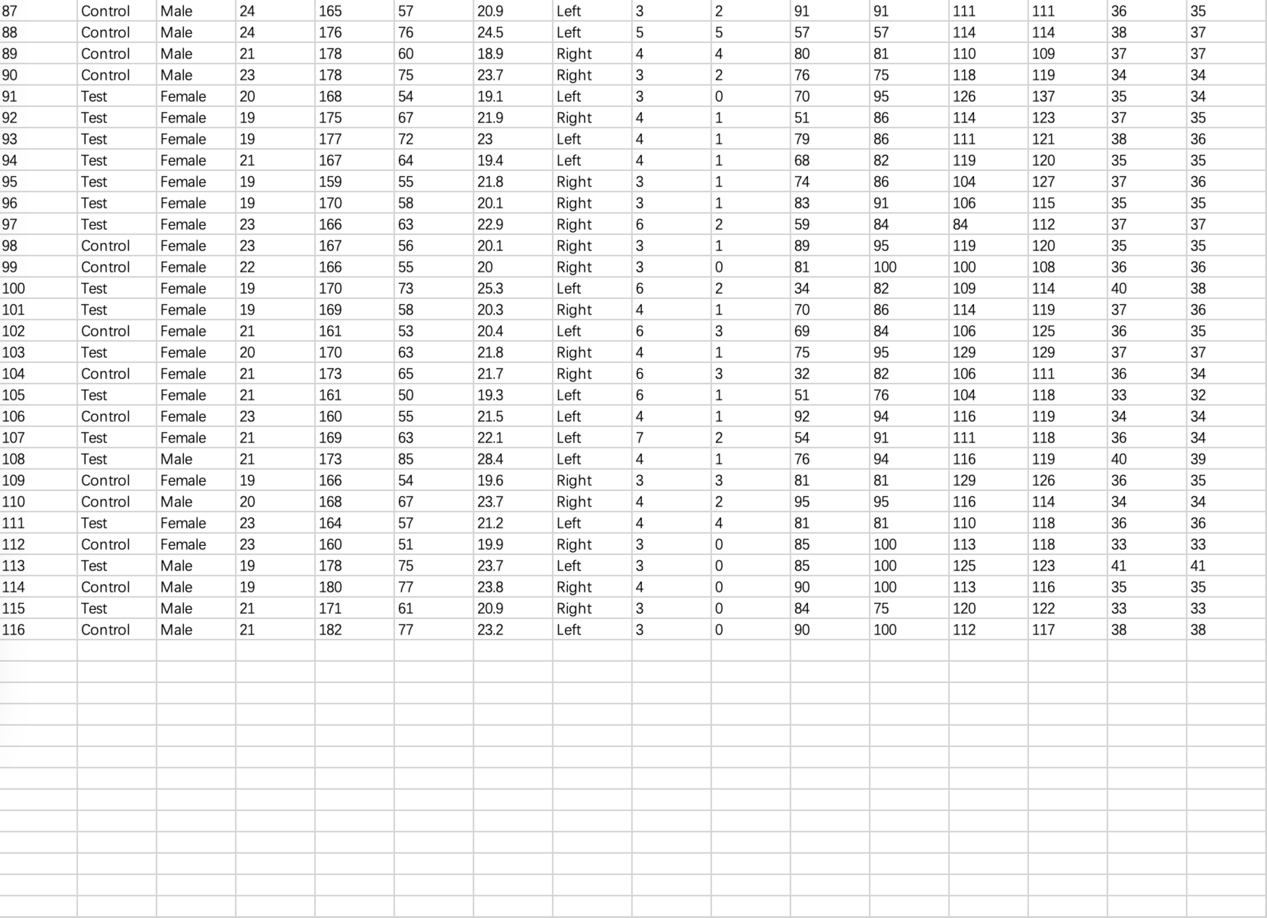

Supplement: Supplementary Materials — Numbers refer to the serial number; Groups refer to the test/control groups; Leg refers to which leg the injured knee belongs to; Age, Height, Weight, and BMI are the real information of the participants; Visual Analogue Scale pain score was regarded as the primary outcome; and the Lysholm, Range of Motion, Circumference were considered the secondary outcomes. Both groups were assessed before the first treatment (referred to before in the picture) and 24 h after the third treatment (referer to after in the picture). [file 6806565.f1.docx]
